# Transcriptomic analysis of human IL‐7 receptor alpha^ low^ and^ high^ effector memory CD8^+^ T cells reveals an age‐associated signature linked to influenza vaccine response in older adults

**DOI:** 10.1111/acel.12960

**Published:** 2019-05-01

**Authors:** Hong‐Jai Park, Min Sun Shin, Minhyung Kim, Joshua B. Bilsborrow, Subhasis Mohanty, Ruth R. Montgomery, Albert C. Shaw, Sungyong You, Insoo Kang

**Affiliations:** ^1^ Department of Internal Medicine Yale University School of Medicine New Haven Connecticut; ^2^ Departments of Surgery and Biomedical Sciences Cedars‐Sinai Medical Center Los Angeles California; ^3^ Samuel Oschin Comprehensive Cancer Institute Cedars‐Sinai Medical Center Los Angeles California

**Keywords:** age, human, IL‐7 receptor alpha, memory CD8^+^ T cells, gene expression, vaccine response

## Abstract

Here, we investigated the relationship of the age‐associated expansion of IL‐7 receptor alpha low (IL‐7Rα^low^) effector memory (EM) CD8^+^ T cells with the global transcriptomic profile of peripheral blood cells in humans. We found 231 aging signature genes of IL‐7Rα^low^ EM CD8^+ ^T cells that corresponded to 15% of the age‐associated genes (231/1,497) reported by a meta‐analysis study on human peripheral whole blood from approximately 15,000 individuals, having high correlation with chronological age. These aging signature genes were the target genes of several transcription factors including MYC, SATB1, and BATF, which also belonged to the 231 genes, supporting the upstream regulatory role of these transcription factors in altering the gene expression profile of peripheral blood cells with aging. We validated the differential expression of these transcription factors between IL‐7Rα^low^ and ^high^ EM CD8^+^ T cells as well as in peripheral blood mononuclear cells (PBMCs) of young and older adults. Finally, we found a significant association with influenza vaccine responses in older adults, suggesting the possible biological significance of the aging signature genes of IL‐7Rα^low^ EM CD8^+ ^T cells. The results of our study support the relationship of the expansion of IL‐7Rα^low^ EM CD8^+^ T cells with the age‐associated changes in the gene expression profile of peripheral blood cells and its possible biological implications.

## 1 INTRODUCTION

Age‐associated changes in different elements of the immune system, including cells, organs, and circulating factors, have been reported (Lee, Shin, & Kang, 2012; Nikolich‐Zugich, 2018). In human T cells, one of the most prominent changes with age is the altered proportion of naïve to memory CD8^+^ T cells that have different cellular profiles. With age, the proportion of memory CD8^+^ T cells expands in peripheral blood while the proportion of naïve CD8^+^ T cells decreases (Hong, Dan, Choi, & Kang, 2004). The expanded memory CD8^+^ T cells with age are typically CD27^−^CD28^−^ (downregulated on antigen‐experienced cells), CCR7^−^ (effector memory cells migrating to inflamed sites), and CD57^+^ (replication senescence marker) (Batliwalla, Rufer, Lansdorp, & Gregersen, 2000). Previously, we measured the expression of IL‐7 receptor alpha chain (IL‐7Rα or CD127) on human CD8^+^ T cells in young and older adults, considering the possible role of this cytokine receptor in expanding memory CD8^+^ T cells with age given its capacity to promote memory T‐cell survival. We found that human effector memory (EM) CD8^+^ T cells, which expand with age, comprise two different subsets of cells expressing IL‐7Rα^low^ and ^high^ with distinct characteristics including the expression of effector molecules, transcription factors, and DNA methylation profiles (Kim, Hong, Dan, & Kang, 2006; Kim, Hwang, Kim, & Kang, 2007; Shin et al., 2015). Having high levels of perforin, granzyme B, IFN‐γ, and TNF‐α, IL‐7Rα^low^ EM CD8^+^ T cells are a highly cytotoxic and pro‐inflammatory subset (Kim et al., 2006, 2007; Shin et al., 2015). However, IL‐7Rα^low^ EM CD8^+^ T cells appear to be replication senescent cells with impaired T‐cell receptor‐mediated proliferation and increased expression levels of senescence markers like CD57 (Kim et al., 2006, 2007). Indeed, older adults have increased numbers of IL‐7Rα^low^ cells compared to young adults, indicating the effect of age on the proportion of this cell subset (Kim et al., 2006). This point is in concordance with the results of a recent study that showed the silencing of the *IL7RA* gene by memory CD8^+^ T cells in humans with aging (Ucar et al., 2017).

A recent meta‐analysis on the global gene expression profile of human peripheral whole blood from ~15,000 individuals identified 1,497 genes that were differentially expressed with chronological age (Peters et al., 2015). A combination of gene clustering and pathway analysis identified several major clusters of the differentially expressed genes (DEGs) which correlated with age. These clusters include T‐ and B‐cell signaling, innate immunity, and genes involved in hematopoiesis, likely reflecting the presence of different types of circulating cells in blood and the possible effect of age on such cells (Peters et al., 2015). However, the contributions of individual types of immune cells to the age‐associated changes in the genomic profile of peripheral blood cells are not yet well defined although their gene expression profiles and functions can be distinct.

Influenza can cause significant morbidity and mortality in the elderly (Thompson et al., 2003). The clinically available influenza vaccine can provide protection against infection and reduce the risk of influenza‐associated hospitalization, especially in children, older adults, and patients with chronic health conditions. However, influenza vaccine responses decline with age as determined by the assay for hemagglutination inhibition (HI) antibody titers (Chen et al., 2009; McElhaney, 2011). Such a finding could be related in part to alterations in the immune system with age (Chen et al., 2009; McElhaney, 2011). A less robust rise in serum HI antibody titers was associated with the expansion of memory CD8^+^ T cells expressing CD45RA or lacking CD28 expression in older adults after influenza vaccination (Goronzy et al., 2001). We also observed the correlation of the frequency of memory CD8^+^ T‐cell subsets including IL‐7Rα^high^ EM CD8^+^ T cells with influenza vaccine‐specific IgG responses in young but not older adults (Kang et al., 2013). These findings suggest the distinct relationships of memory CD8^+^ T‐cell subsets with influenza vaccine responses in young and older adults.

Here, we investigated the possible relationship of this age‐associated expansion of IL‐7Rα^low^ EM CD8^+^ T cells with the global transcriptomic profile of peripheral blood cells in humans. Crossing DEGs in IL‐7Rα^low^ EM CD8^+^ T cells against age‐associated genes from human peripheral blood (Peters et al., 2015) revealed an age‐associated gene expression signature of the IL‐7Rα^low^ EM CD8^+^ T cells. We selected a set of genes highly correlating with age and their potential transcriptional regulators using bioinformatic analysis. The differential expression of these genes in peripheral blood mononuclear cells (PBMCs) was validated in our cohorts of young and older subjects. Also, we showed the possible relationship of such genes with influenza vaccine responses in two distinct cohorts of older adults.

## 2 RESULTS

### 2.1 IL‐7Rα^low^ and^ high^ EM CD8^+^ T cells have distinct global gene expression profiles

**Figure 1:**
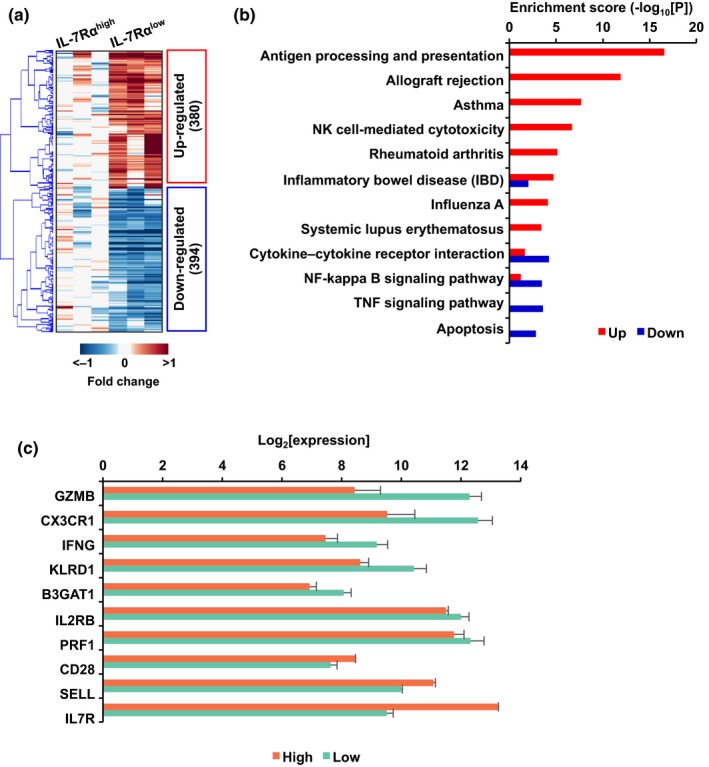
IL‐7Rα^low^ and ^high^ effector memory CD8^+^ T cells have distinct global gene expression profiles. Gene expression microarray analysis was done on IL‐7Rα^low^ and ^high^ effector memory (EM) CD8^+^ T cells purified from PBMCs of healthy donors (*n* = 3). (a) Heatmap displays of the differential expression of 774 genes. Red and blue indicate up‐ and downregulations, respectively. (b) Enriched Kyoto Encyclopedia of Genes and Genomes (KEGG) pathways by 380 upregulated and 394 downregulated genes in IL‐7Rα^low^ cells compared to IL‐7Rα^high^ cells. (c) Bar plot shows expression levels of the genes encoding the molecules known to be up or downregulated in IL‐7Rα^low^ cells compared to IL‐7Rα^high^ cells

In humans, the proportions of peripheral IL‐7Rα^low^ and ^high^ EM CD8^+^ T cells with distinct cellular phenotypes and functions alter with age (Kim et al., 2006, 2007; Shin et al., 2015). However, their global gene expression profiles had not previously been defined. Here, we found 774 genes that were differentially expressed between IL‐7Rα^low^ and ^high^ EM CD8^+^ T cells by performing global gene expression analysis (Figure 1a and Table S1). Functional enrichment analysis of the DEGs revealed the enriched Kyoto Encyclopedia of Genes and Genomes (KEGG) pathways related to natural killer (NK) cell‐mediated cytotoxicity, antigen processing and presentation, and cytokine/chemokine pathways as well as autoimmune diseases and influenza pathways (Figure 1b). In concordance with our published studies (Kim et al., 2006; Lee et al., 2014), the results of the gene expression array showed increased expression levels of the *GZMB*, *CX3CR1*, *IFNG*, *KLRD1*, *B3GAT1* (*CD57)*, *IL2RB,* and *PRF1* genes in IL‐7Rα^low^ EM CD8^+^ T cells while the expression levels of the *CD28*, *SELL,* and *IL7R* genes were lower in the same cells compared to IL‐7Rα^high^ EM CD8^+^ T cells (Figure 1c).

### 2.2 An age‐associated signature exists in human IL‐7Rα^low^ EM CD8^+^ T cells

**Figure 2:**
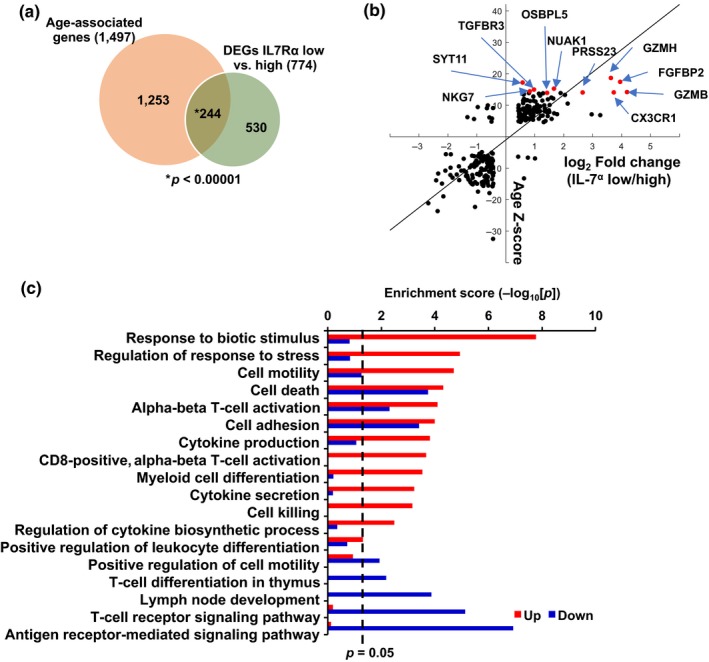
IL‐7Rα^low^ effector memory CD8^+^ T cells express a set of aging signature genes that are associating with human chronological age. (a) Venn diagram depicts overlapping genes between the differentially expressed genes (DEGs) of IL‐7Rα^low^ versus ^high^ effector memory (EM) CD8^+^ T cells and age‐associated genes identified from a meta‐analysis of whole‐blood global gene expression microarrays on approximately 15,000 human subjects. *p* value indicates significance of the number of overlapping genes based on a random permutation strategy (see Methods). (b) Scatter plot of the overlapping genes shows a correlative relationship between log2‐fold changes of IL‐7Rα^low^ versus ^high^ EM CD8^+^ T cells and age‐associated *z* scores from Figure 1 and the meta‐analysis in (a), respectively. Dots on the plot indicate individual genes. Genes with the top 10 highest age‐associated *z* scores are highlighted with labels and arrows. (c) Bar plot shows enriched biological processes of up and downregulated aging signature genes in IL‐7Rα^low^ EM CD 8^+^ T cells in comparison with IL‐7Rα^high^ EM CD8^+^ T cells

A recent meta‐analysis on the global gene expression profile of human peripheral whole blood from approximately 15,000 individuals identified 1,497 genes that correlated with chronological age (Peters et al., 2015). We determined the number of the 774 DEGs between IL‐7Rα^low^ and ^high^ EM CD8^+^ T cells that corresponded to these 1,497 genes. About one third (244/774) of the DEGs between the two cell subsets corresponded to 15% (244/1,497) of the age‐associated genes (Figure 2a). We selected 231 out of the 244 genes based on the correlation of the fold changes between IL‐7Rα^low^ and ^high^ EM CD8^+^ T cells with the age *z* scores (Figure 2b). The fold changes and age *z* scores of the 231 genes were highly correlated (Pearson's *r* = 0.81 and *p* < 0.001, as shown in Figure 2b where the top 10 highest age *z* score genes were indicated in red dots). These 231 genes were referred to as the aging signature genes of IL‐7Rα^low^ EM CD8^+^ T cells (Table S2). Functional enrichment analysis of up‐ and downregulated genes among these aging signature genes demonstrated enrichment in the biological processes related to T cells, including T‐cell receptor signaling, differentiation, and activation, as well as to cell death, adhesion, cytokine production, and cell killing (Figure 2c).

### 2.3 Transcriptional regulatory network analysis shows the unique gene expression profile of the aging signature genes

**Figure 3:**
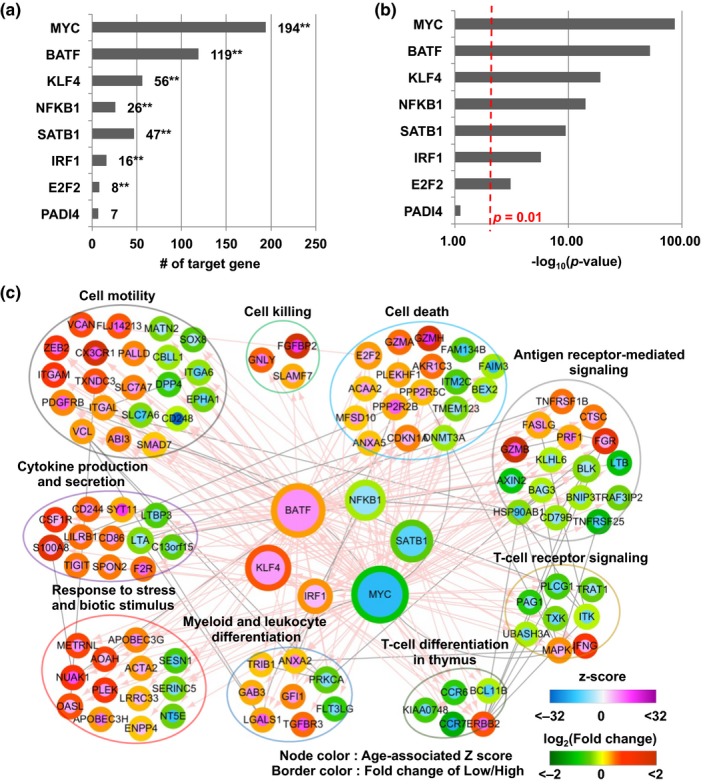
A network model describes interactions between key transcription factors and the aging signature genes regulated by these transcription factors. (a,b) Bar plots display the numbers and −Log10 *p* values of the target genes for the individual transcription factors. *p* values were computed using Fisher's exact test. (c) A network model describes regulatory relationships between transcription factors and functional modules. The relationships of the age‐associated *z* scores of individual genes with the expressional fold changes of these same genes between IL‐7Rα^low^ and ^high^ cells are shown as the nodes and border colors, respectively. Gray solid and pink arrowed edges represent protein–protein and transcription–target interactions respectively

We next explored the possible involvement of upstream transcriptional regulators in the expression of the 231 aging signature genes of IL‐7Rα^low^ EM CD8^+^ T cells. We found eight transcription factor encoding genes including *MYC*, *BATF*, *KLF4*, *NFKB1*, *SATB1*, *IRF1*, *E2F2,* and *PADI4* that were differentially expressed between IL‐7Rα^low^ and ^high^ EM CD8^+^ T cells. We interrogated the relationship of these transcription factors with the aging signature genes using Master Regulator Analysis of Transcription Factors (Lefebvre et al., 2010). Six of the eight transcription factors, including MYC, BATF, KLF4, NFKB1, SATB1, and IRF1*,* had more than 10 targets in the aging signature genes with significant transcriptional regulatory potential (*p* < 0.01; Figure 3a,b). Using the transcriptional regulatory interactions between the six transcription factors and the aging signature genes (see Methods), we reconstructed a transcriptional regulatory network describing the relationships of these six transcription factors with their targets in the aging signature genes as well as the functional associations of the target genes (Figure 3c). The target genes in the network can be grouped into nine functional modules (i.e., cell killing, cell death, cell motility, antigen receptor‐mediated signaling, cytokine production and secretion, response to stress and biotic stimulus, myeloid and leukocytes differentiation, T‐cell differentiation in thymus, and T‐cell receptor signaling) based on the functional annotations from gene ontology biological processes and KEGG pathways in david software. These findings suggest that the transcription factors encoded by *MYC*, *BATF, SATB1*, *KLF4, IRF1*, and *NFKB1* could be the major upstream regulators for the age‐associated signature genes of IL‐7Rα^low^ EM CD8^+^ T cells.

### 2.4 The expression levels of the *MYC*, *BATF,* and *SATB1* genes are altered in IL‐7Rα^low^ EM CD8^+^ T cells and PBMCs of older adults as compared to IL‐7Rα^high^ EM CD8^+^ T cells and PBMCs of young adults

**Figure 4:**
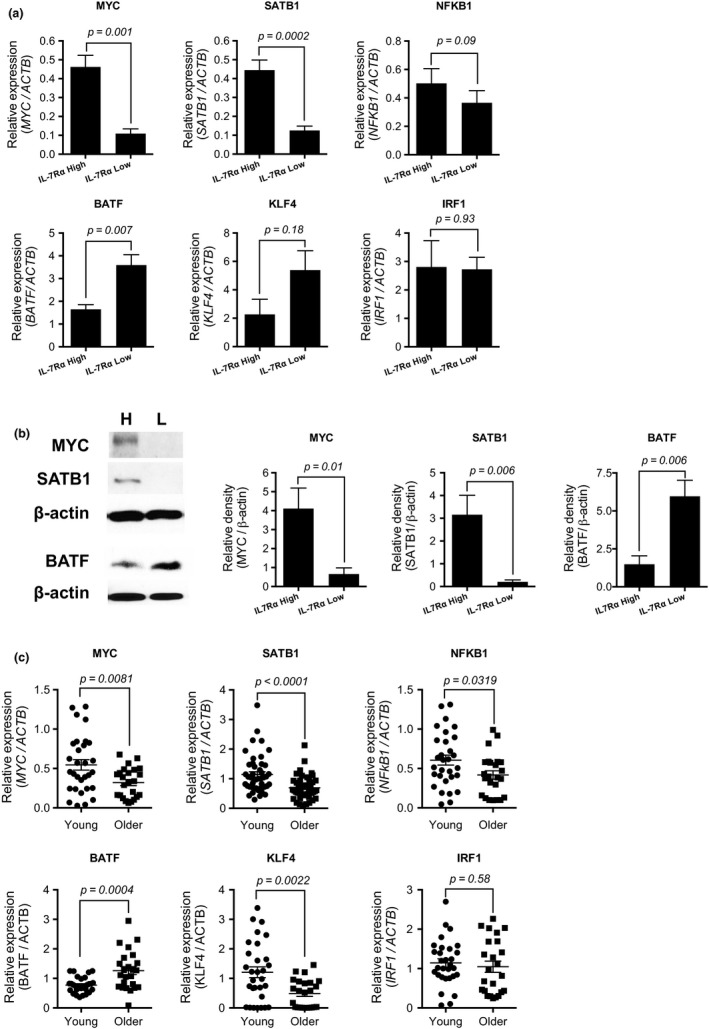
IL‐7Rα^low^ effector memory CD8^+^ T cells have differential expression of the transcription factors MYC, SATB1, and BATF that change in peripheral blood mononuclear cells with age. (a) qPCR analysis of the indicated genes in IL‐7Rα^high^ and ^low^ effector memory CD8^+^ T cells purified from PBMCs of 5–10 mixed‐age human subjects. (b) Western blot analysis of MYC, SATB1, and BATF in IL‐7Rα^high^ (H) and ^low^ (L) effector memory CD8^+^ T cells purified from PBMCs of 6–9 mixed‐age human subjects. Representative blots and the relative density of each molecule compared to β‐actin are shown. (c) qPCR analysis of the indicated genes in peripheral blood of young (*n* = 30) and older (*n* = 24) adults. Bars and error bars indicate the means ± and *SEM* (a,b). Bars indicate the means (c). *p* values were obtained by the paired (a,b) and unpaired (c) *t* tests

We next confirmed the differential expression of *MYC*, *BATF1,* and *SATB1* between IL‐7Rα^low^ and ^high^ EM CD8^+^ T cells from mixed‐age adult donors (see Experimental Procedures) using qPCR (Figure 4a). Although there was a trend toward decreased expression of *NFKB1* in IL‐7Rα^low^ EM CD8^+^ T cells compared to IL‐7Rα^high^ EM CD8^+^ T cells, this difference was not statistically significant. The expression of *KLF4* appeared to be higher in IL‐7Rα^low^ EM CD8^+^ T cells than in IL‐7Rα^high^ EM CD8^+^ T cells, though this also did not reach the level of statistical significance. In concordance with the results of qPCR, IL‐7Rα^low^ EM CD8^+^ T cells had lower levels of MYC and SATB1 and higher levels of BATF protein expression compared to IL‐7Rα^high^ EM CD8^+^ T cells in mixed‐age adult donors (Figure 4b). We also analyzed the expression levels of these genes in PBMCs of young (age ≤ 40) and older (age ≥ 65) adults using qPCR. Older adults had decreased levels of *MYC*, *SATB1, and NFKB1* and increased levels of *BATF* compared to young adults (Figure 4c), which concurs with previous results of global gene expression meta‐analysis in human aging (Peters et al., 2015). These changes could be related in part to the age‐associated expansion of IL‐7Rα^low^ EM CD8^+^ T cells in peripheral blood. Although the expression of *KLF4* appeared to be increased in IL‐7Rα^low^ EM CD8^+^ T cells, the levels of this gene were lower in older adults than in young adults, suggesting that other types of immune cells might be related to the latter finding.

### 2.5 The expression levels of *granzyme H (GZMH), fibroblast growth factor binding protein 2 (FGFBP2),* and *CX3CR1* genes are altered in IL‐7Rα^low^ EM CD8^+^ T cells and PBMCs of older adults as compared to IL‐7Rα^high^ EM CD8^+^ T cells and PBMCs of young adults

**Figure 5:**
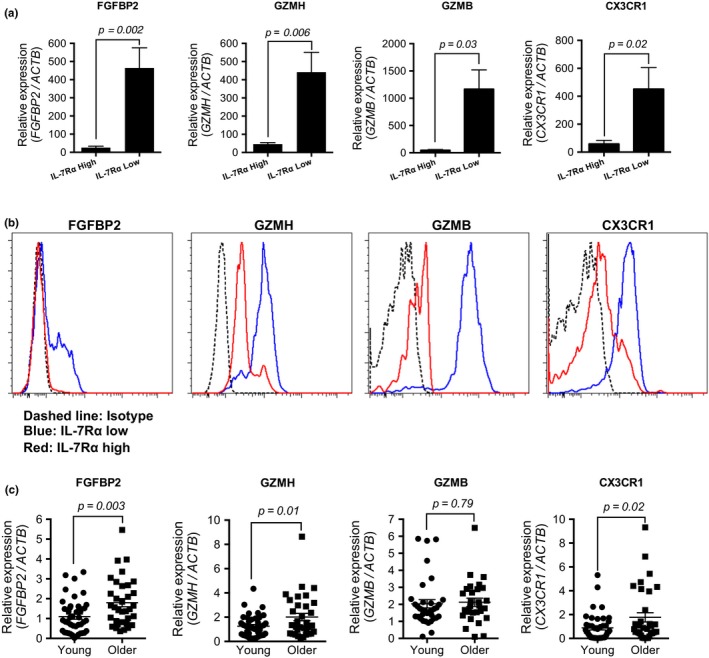
IL‐7Rα^low^ effector memory CD8^+^ T cells have differential expression of FGFBP2, GZMH, and CX3CR1 that change in peripheral blood mononuclear cells with age. (a) qPCR analysis of the indicated genes in IL‐7Rα^high^ and ^low^ effector memory CD8^+^ T cells purified from PBMCs of 5–10 mixed‐age human subjects. Bars and error bars indicate means ± and *SEM*. (b) Flow cytometric analysis of fibroblast growth factor binding protein 2 (FGFBP2), granzyme H (GZMH), granzyme B (GZMB), and CX3CR1 in IL‐7Rα^high^ and ^low^ effector memory CD8^+^ T cells of PBMCs. Representative data from 5 to 10 mixed‐age human subjects. (c) qPCR analysis of indicated genes in peripheral blood of young (*n* = 35–43) and older (*n* = 28–33) adults. Bars indicate the means. *p* values were obtained by the paired (a) and unpaired (c) *t* tests

Among the 231 age‐associated signature genes of IL‐7Rα^low^ EM CD8^+^ T cells, the genes encoding the cytotoxic molecules GZMH, GZMB, FGFBP2, and the chemokine receptor CX3CR1 were the ones with the highest levels of age‐associated *z* scores and fold changes of the gene expression between IL‐7Rα^low^ and ^high^ EM CD8^+^ T cells (Table 1). This finding is consistent with our previous studies showing increased gene and/or protein expression of GZMB and CX3CR1 by IL‐7Rα^low^ EM CD8^+^ T cells compared to IL‐7Rα^high^ EM CD8^+^ T cells (Shin et al., 2015). Similarly, we also noted higher levels of FGFBP2 and GZMH gene and protein expression in IL‐7Rα^low^ EM CD8^+^ T cells than in IL‐7Rα^high^ EM CD8^+^ T cells in mixed‐age adult donors (Figure 5a,b). We next analyzed these genes in PBMCs of young and older adults using qPCR. Older adults had increased levels of FGFBP2, GZMH, and CX3CR1 in PBMCs compared to young adults (Figure 5c). These findings again support the possible relationship of the age‐associated expansion of IL‐7Rα^low^ EM CD8^+^ T cells with the changes in the genomic profile of peripheral blood.

**Table 1:** The top 10 genes with the highest age‐associated *z* scores among the aging signature genes

Entrez ID	Symbol	Age‐associated *z* score	Low/high
2999	GZMH	18.681	3.63
83888	FGFBP2	17.449	3.95
23208	SYT11	17.225	0.58
9891	NUAK1	15.281	1.66
7049	TGFBR3	14.997	0.97
4818	NKG7	14.434	0.83
3002	GZMB	14.19	4.19
1524	CX3CR1	14.094	3.73
11098	PRSS23	14.071	2.65
114879	OSBPL5	13.939	1.43

### 2.6 Aging signature genes of IL‐7Rα^low^ EM CD8^+^ T cells predict influenza vaccine responses in older adults

**Figure 6:**
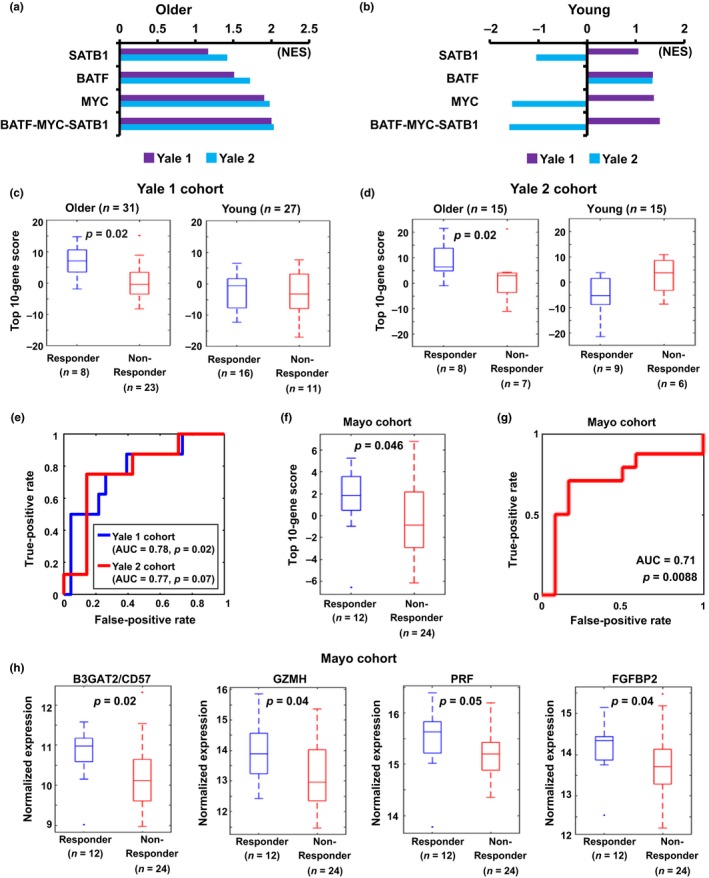
Association of the aging signature genes of IL‐7Rα^low^ effector memory CD8^+^ T cells with vaccine responses in young and older adults. (a,b) Bar plots display normalized enrichment scores (NES) of the target genes of the indicated transcription factors in the Yale 1 and Yale 2 cohorts. Gene Set Enrichment Analysis (GSEA) was performed in older (a, age ≥ 65) and young (b, age ≤ 35) adults, separately. NES indicate the distribution of genes in the network model across a list of genes ranked by signal‐to‐noise ratio between two groups. Higher NES indicates a shift of genes belonging to the network toward either end of the ranked list, representing up (positive NES) or down (negative NES) regulation (see Figure S1). (c,d) Boxplots display top 10 gene scores in vaccine responders and nonresponders of older and young adults from the Yale 1 (c) and Yale 2 (d) cohorts. (e) Receiver operating characteristic (ROC) curves show the performance of a logistic regression model examining the relationship between the top 10 gene score and vaccine responses in older adults of the Yale 1 and Yale 2 cohorts. (f) Boxplot depicts top 10 gene scores in vaccine responders and nonresponders of older adults (age ≥ 65) from the Mayo cohort. (g) ROC curve shows performance of the regression model in older adults (age ≥ 65) of the Mayo cohort. (H) Boxplots display the differential expression of indicated genes associated with immunosenescence (B3GAT2/CD57) and cytotoxicity (GZMH, PRF, and FGFBP2) of T cells between vaccine responders and nonresponders in older adults of the Mayo cohort. Vaccine responses were determined by hemagglutination inhibition (HAI) antibody responses (Yale 1 and Yale 2 cohorts) or a combination of HAI antibody responses and B‐cell Enzyme Linked ImmunoSpot (ELISPOT) count values (Mayo cohort). The boxes show the 25th‐75th percentile range and the center line is the median. Whiskers show 1.5 times IQR from the 25th or 75th percentile values. Data points beyond the whiskers are displayed using dots. *p*‐values were obtained by the Wilcoxon rank‐sum statistics (c,d,f,h)

The results of our pathway enrichment analysis showed the significant association of the influenza A pathway with the upregulated DEGs in IL‐7Rα^low^ EM CD8^+^ T cells (Figure 1b). Influenza vaccine responses decline with age (Chen et al., 2009; McElhaney, 2011). We thus explored the possible relationship of the aging signature genes of IL‐7Rα^low^ EM CD8^+^ T cells with influenza vaccine responses in young and older adults using publicly available gene expression data deposited in the Gene Expression Omnibus (GEO) and ImmPort website. For this, we analyzed three independent cohorts referred to as Yale 1, Yale 2, and Mayo comprised of young and older adults with influenza vaccine responses and gene expression data (see Methods). The Yale 1 and Yale 2 cohorts were from a study where PBMCs of young (age ≤ 35) and older (age ≥ 65) adults were analyzed for genomewide gene expression before and after influenza vaccination (Thakar et al., 2015), while the Mayo cohort was from a study where PBMCs of adults (50 ≤ age ≤ 74) were analyzed by performing RNA sequencing before and after influenza vaccination (Ovsyannikova et al., 2016). We first tested whether the target genes of *MYC*, *BATF* and *SATB1* and all of the three transcription factors (Table S4), which were in the network model shown in Figure 3c, were enriched in vaccine responders using the Gene Set Enrichment Analysis (GSEA) method (Subramanian et al., 2005). The genes targeted by the three transcription factors in the network model were all significantly enriched in older responders in both Yale data sets. This enrichment pattern was noticed in the young responders of the Yale 1 but not the Yale 2 cohort except the target genes of BATF (Figure 6a,b and Figure S1). These findings suggest that MYC, BATF, and SATB1 are part of the upstream factors determining immune responses to influenza vaccine through regulating downstream gene expression in CD8^+^ T cells although such a program may operate distinctively in young and older adults.

We further investigated whether a subset of aging signature genes could predict influenza vaccine responses in young and older groups. For this, we selected genes with the top 10 highest age *z* scores that were upregulated at twofold or greater levels in IL‐7Rα^low^ EM CD8^+^ T cells (Table 1 and Figure 2b). Based on the expression levels of these genes, we computed a top 10 gene score for each subject using the weighted *z* score method (Levine et al., 2006). In older adults of the Yale 1 cohort, the top 10 gene scores were significantly higher in vaccine responders than in nonresponders (Wilcoxon rank‐sum test, *p* = 0.02; Figure 6c). However, in the young adults of the same cohort, the top 10 gene scores were not different between vaccine responders and nonresponders. The same findings were observed in the Yale 2 cohort (Figure 6d). We next tested whether the top 10 gene score was predictive of the vaccine response in the older group. A logistic regression model with the top 10 genes was trained using the Yale 1 cohort. We then tested the model performance for the prediction of responder and nonresponder in the older group, showing good performance in both the Yale 1 (area under the curve (AUC) = 0.78, *p* = 0.02) and Yale 2 (AUC = 0.77, *p* = 0.07) cohorts (Figure 6e). To further validate these findings and avoid potential biases from the cohorts, we applied the regression model to the Mayo cohort (Voigt et al., 2018) and checked its performance for the separation of responders and nonresponders. The results of this analysis showed a similar level of significance in the top 10 gene score and classification performance for older (age ≥ 65) vaccine responders versus nonresponders as found in the Yale cohorts (Figure 6f,g). In addition, the expression levels of *B3GAT2* (CD57), *GZMH*, *PRF,* and *FGFBP2* genes were significantly lower in nonresponders than in responders (Figure 6h). Collectively, these data suggest that the aging gene signature regulated by *BATF‐MYC‐SATB1* in IL‐7Rα^low^ EM CD8^+^ T cells is predictive of influenza vaccine responses in older adults.

## 3 DISCUSSION

Aging affects multiple organ systems at the levels of genes, cells, and tissues. Age‐associated alterations in gene expression have been systemically investigated in organisms like *C. elegans* and mice using genomewide approaches (Jones et al., 2001; Weindruch, Kayo, Lee, & Prolla, 2001). A recent whole‐blood gene expression meta‐analysis in about 15,000 individuals of European ancestry identified 1,497 genes that were differentially expressed depending on chronological age (Peters et al., 2015). The clusters of coexpressed genes which were correlated positively or negatively with age contained molecules related to immunity. We observed that about 15% (231/1,497 genes) of these age‐associated genes corresponded to one third of the DEGs in IL‐7Rα^low^ EM CD8^+^ T cells in comparison with IL‐7Rα^high^ EM CD8^+ ^T cells. This finding is remarkable since memory CD8^+^ T cells account for only 1%–7% of total leukocytes. With age, IL‐7Rα^low^ EM CD8^+^ T cells increase while IL‐7Rα^high^ EM CD8^+^ T cells decrease in peripheral blood (Kim et al., 2006). Thus, a possible explanation for our finding could be a disproportional alteration in the frequency of IL‐7Rα^low^ and ^high^ EM CD8^+^ T cells compared to other types of immune cells. Alternatively, but not mutually exclusive, some of the DEGs in IL‐7Rα^low^ EM CD8^+^ T cells could change with aging at the cellular level. A recent study identified the silencing of *IL7R* and IL‐7 signaling pathway genes in human peripheral blood cells as potential biomarkers for aging based on combined analyses of genomewide chromatic accessibility and transcriptome (Ucar et al., 2017). Of interest, such a gene signature stemmed from the memory CD8^+^ T cells (Ucar et al., 2017). These findings are consistent with the results of our study showing the implication of IL‐7Rα^low^ EM CD8^+^ T‐cell expansion in changing the genomic profile of peripheral blood with aging as well as with our previous work demonstrating the distinct pattern of genomewide DNA methylation in IL‐7Rα^low^ EM CD8^+^ T cells which expand with age (Kim et al., 2006; Shin et al., 2015).

A large number of the aging signature genes of IL‐7Rα^low^ EM CD8^+^ T cells are the target genes of several transcription factors including MYC, SATB1, and BATF. Of interest, 194 of the 231 aging signature genes of IL‐7Rα^low^ EM CD8^+^ T cells are MYC target genes. Considering that MYC regulates the expression of about 15% of all genes (Gearhart, Pashos, & Prasad, 2007), the MYC target genes are highly enriched among the aging signature genes of IL‐7Rα^low^ EM CD8^+^ T cells. Studies have reported on the role of MYC in regulating CD8^+^ T‐cell immunity, mostly using animal models. Retroviral overexpression of MYC in mice promoted the generation of memory CD8^+^ T cells (Haque et al., 2016). *MYC* haploinsufficient (*Myc* ^±^) mice with reduced levels of whole‐body MYC had increased longevity and less immunosenescence, including the increased ratio of naïve to memory T cells, compared to wild‐type mice (Hofmann et al., 2015). However, studies reported decreased *MYC* gene expression in T cells of older adults and aged mice with reduced cell proliferation (Buckler, Vie, Sonenshein, & Miller, 1988; Gamble, Schwab, Weksler, & Szabo, 1990). In concordance with these findings, we noticed decreased MYC expression in IL‐7Rα^low^ EM CD8^+^ T cells, which are known to have impaired TCR‐mediated proliferation and expand with age (Kim et al., 2006), in mixed‐age adults as well as decreased expression of the *MYC* gene in PBMCs of older adults.

The basic leucine zipper transcription factor BATF is known to regulate differentiation of effector CD8^+^ T cells (Kurachi et al., 2014; Quigley et al., 2010). BATF expression is increased in exhausted T cells during chronic viral infection in humans and in mice (Quigley et al., 2010). Mice deficient of BATF in CD8^+^ T cells had impaired expansion of effector CD8^+^ T cells after encountering antigen (Kurachi et al., 2014). Also, BATF promoted T‐bet and Blimp‐1, which are both highly expressed in terminally differentiated CD8^+^ T cells like IL‐7Rα^low^ EM CD8^+^ T cells (Lee et al., 2014). A recent human study reported increased chromatin accessibility and gene expression of *BATF* in memory CD8^+^ T cells (Moskowitz et al., 2017). Of note, the expression levels of the BATF target genes, identified by knocking down human cell lines, were increased in human T cells with aging (Moskowitz et al., 2017). We observed increased expression of BATF in IL‐7Rα^low^ EM CD8^+^ T cells compared to IL‐7Rα^high^ EM CD8^+^ T cells in mixed‐age donors. Also, PBMCs of older adults had higher levels of *BATF* expression than those of young adults. Taken together, these findings support the possible implication of BATF in differentially regulating gene expression of human IL‐7Rα^low^ and ^high^ EM CD8^+^ T cells as well as in altering the gene expression profiles of PBMCs of older adults as an upstream regulator.

SATB1 organizes chromatin architecture and regulates the expression of multiple genes by recruiting chromatin remodeling factors, corepressors, and coactivators (Kohwi‐Shigematsu et al., 2013; Satoh et al., 2013). The role of SATB1 in mature CD8^+^ T cells is largely unknown except for its suppressive effect on PD‐1 expression in murine CD8^+^ T cells (Stephen et al., 2017). Our study demonstrated substantially decreased SATB1 in human IL‐7Rα^low^ EM CD8^+^ T cells, which are highly cytotoxic, inflammatory, and replication senescent compared to IL‐7Rα^high^ EM CD8^+^ T cells. In addition, we observed decreased *SATB1* in human PBMCs with age, which concurs with the finding of decreased *SATB1* in human whole blood and CD8^+^ T cells with age (Peters et al., 2015). The decreased expression of the *SATB1* gene in IL‐7Rα^low^ EM CD8^+^ T cells could be related to decreased chromatin accessibility and increased DNA methylation of the gene since these mechanisms have been suggested to regulate *SATB1* expression in CD8^+^ T cells, especially in the context of aging (Ucar et al., 2017).

We observed that many of the 231 aging signature genes of IL‐7Rα^low^ EM CD8^+^ T cells are those related to CD8^+^ T‐cell activation, cell killing, adhesion, and mobility. In fact, the expression fold changes and age‐associated *z* scores of the *GZMH*, *GZMB*, *FGFBP2,* and *CX3CR1* genes were remarkably high and strongly correlated. We found increased levels of *GZMH*, *FGFBP2,* and *CX3CR1* in PBMCs of older adults compared to young adults. The increased *GZMH* expression in PBMCs of older adults can be related to the increased chromatin accessibility to this gene with age (Ucar et al., 2017). In fact, the DNA methylation‐mediated gene regulatory mechanism could account for the altered expression of *GZMH* and *CX3CR1* in PBMCs with age since our published study found decreased DNA methylation, which is associated with increased gene expression, in the *CX3CR1* and *GZMH* genes in IL‐7Rα^low^ EM CD8^+^ T cells compared to IL‐7Rα^high^ EM CD8^+^ T cells (Shin et al., 2015). Of note, our gene expression array data showed higher levels of the transcriptional repressor *growth factor independent‐1* (*GFI1*) in IL‐7Rα^low^ EM CD8^+^ T cells compared to IL‐7Rα^high^ EM CD8^+^ T cells (Table S1). This finding is in line with the results of a previous study reporting the role of GFI1 in suppressing IL‐7Rα expression in mouse lymph node CD8^+^ T cells (Park et al., 2004).

Our pathway enrichment analysis showed the significant association of the influenza A pathway with the upregulated DEGs in IL‐7Rα^low^ EM CD8^+^ T cells. We observed that older adults who developed immune responses to influenza vaccine had increased aging signature genes including those targeted by *MYC*, *BATF,* and/or *SATB1* compared to older adults who were vaccine nonresponders. However, this phenomenon was not found among young adults. Although the exact mechanism for these findings has yet to be elucidated, it could be related to the cellular characteristics of IL‐7Rα^low^ EM CD8^+^ T cells which express high levels of chemokine receptors (e.g., CX3CR1, CXCR1), inflammatory cytokines (IFN‐γ, TNF‐α), and cytotoxic molecules (GZMA, B, H, FGFBP2, perforin, and granulysin) (Shin et al., 2015). By expressing these chemokine receptors, these cells can migrate rapidly to a vaccine‐injected site and secrete IFN‐γ and TNF‐α which may enhance the local immune response to the vaccinated antigen like an adjuvant. We understand that this notion is speculative and difficult to test in vivo in humans. Anti‐TNF‐α treatment can reduce influenza vaccine responses (Rubin et al., 2014). Also, we showed that soluble factors from IL‐7Rα^low^ but not from IL‐7Rα^high^ EM CD8^+^ T cells induced upregulation of fractalkine (CX3CR1 ligand) and IL‐8 (CXCR1 ligand) in human endothelial cells and neutrophils dependently of TNF‐α and IFN‐γ (Shin et al., 2015) (CXCR1 data, Shin et al., unpublished observation). In fact, monocytes that can differentiate dendritic cells express high levels of CX3CR1 and CXCR1 (Geissmann, Jung, & Littman, 2003). The production of granzyme B from influenza virus‐stimulated PBMCs predicted protection from influenza vaccination (McElhaney et al., 2009). In a study where RNA‐seq analysis was done on PBMCs of subjects from 50 to 74 years of age before and after influenza vaccination, gene clusters enriched for NK and T‐cell activities were associated with influenza virus antibody responses as measured by HAI and virus‐neutralizing influenza A/H1N1 antibody (VNA) titers (Voigt et al., 2018). The top five genes of each of the two clusters (NK cell activity; NK and T‐cell activity) that were most closely correlated with influenza antibody titers had seven genes (*SPON2, AKR1C3, PRF1, FCRL6, GZMA, CSTK, NKG7*) which belong to the aging signature genes of IL‐7Rα^low^ EM CD8^+^ T cells.

Taken together, the results of our study demonstrate the relationship of human IL‐7Rα^low^ EM CD8^+^ T cells with the changes in the global gene expression profile of peripheral blood with age by identifying the aging signature genes of these cells. The expression of such genes is likely regulated by a set of transcription factors including MYC, SATB1, and BATF. The aging signature genes of IL‐7Rα^low^ EM CD8^+^ T cells are linked to influenza vaccine responses as determined by measuring antibody production, highlighting the possible biological significance of altered expression of these genes with age.

## 4 EXPERIMENTAL PROCEDURES

### 4.1 Human subjects

Healthy young subjects ≤40 years of age (*n* = 46) and older subjects ≥65 years of age (*n* = 40) were recruited for this study (mean age ± *SD*, 25.5 years ± 2.83 and 72 years ± 6.65, respectively). The gender distribution was not different between the two groups (F:M, 22:21 and 18:22, respectively, for young and older adult groups, *p* = 0.8406 by the chi‐square test). Additional mixed‐age healthy adults (mean age ± *SD*, 43.5 years ± 14.9, and gender ratio F:M, 10:5) were recruited to compare the characteristics of IL‐7Rα^low^ and ^high^ EM CD8^+^ T cells within a single subject using qPCR, flow cytometry, and Western blot. For gene expression microarray analysis, peripheral blood was obtained from three healthy adults (ages of 38, 45, and 46, respectively). Individuals who were on immunosuppressive drugs or carried a medical condition potentially affecting the immune system, including cancer and autoimmunity, were excluded (Kim et al., 2006). Informed consent was obtained from all subjects. This work was approved by the institutional review committee of Yale University.

### 4.2 Cells, flow cytometry, and cell sorting

Mononuclear cells were prepared from peripheral blood on Ficoll‐Paque (GE Healthcare) gradients as previously done (Shin et al., 2015). Peripheral blood mononuclear cells (PBMCs) were labeled with antibodies to APC‐cyanin 7 (Cy7)‐CD3, Pacific Blue–CD8α, PE–Cy5–CD45RA, PE‐Cy7‐CCR7 (all from BD Biosciences), FITC‐IL‐7Rα (R&D Systems), and PE‐CX3CR1 (BioLegend). Some cells were fixed and permeabilized using Cytofix/Cytoperm solution (BD Biosciences) and additionally stained with antibodies to PE‐FGFBP2, PE‐GZMB, and biotinylated GZMH (all from R&D Systems). Biotinylated GZMH antibodies were then labeled with Pacific Blue conjugated anti‐goat IgG (Biolegend) secondary antibodies. Stained cells were analyzed using an LSRII® flow cytometer (BD Biosciences) and FlowJo software (TreeStar). For FACS sorting of IL‐7Rα^low^ and ^high^ EM CD8^+^ T cells, freshly isolated PBMCs were stained with antibodies to APC‐Cy7‐CD16, Pacific Blue–CD8α, PE–Cy5–CD45RA, PE‐Cy7‐CCR7, and FITC‐IL‐7Rα antibodies. Stained cells were sorted into IL‐7Rα^low^ and ^high^ EM (CCR7^−^) CD8^+^ T cells using a FACSAria® (BD Biosciences). The purity of cells was >97%.

### 4.3 Microarray analysis

Total RNA prepared using an RNeasy mini kit (Qiagen) (RNA integrity numbers ~10) was amplified and hybridized to an Illumina HumanHT‐12v4 Expression BeadChip (Illumina). The probe intensities were normalized using the quantile normalization procedure (Bolstad, Irizarry, Astrand, & Speed, 2003). The data were deposited in the GEO database (GSE97550). Differentially expressed genes (DEGs) between IL‐7Rα^low^ and ^high^ EM CD8^+^ T cells were identified using the Stouffer's method where the overall *p*‐values for individual genes were computed by combining the *p*‐values obtained by the two‐tailed *t* test and log2 median ratio test (Hwang et al., 2005). The false discovery rate (FDR) was computed by adjusting the overall *p*‐value using the Storey's method (Storey & Tibshirani, 2003). The DEGs were selected based on the false discovery rate (FDR) < 0.05 and absolute log2‐fold changes >0.4 which was determined by fitting a Gaussian distribution to the log2‐fold changes of randomly permuted samples and calculating the 2.5th percentile (*α* = 0.05). Functional enrichment analysis was performed using the Database for Annotation, Visualization, and Integrated Discovery (DAVID) 6.8. The cellular processes represented by the genes were identified by the Gene Ontology (GO) biological processes (*p* < 0.05).

### 4.4 Identification of key transcription factors

Transcription factors and their target interaction data were collected from publicly available databases including TRED, EEDB, MSigDB, Amadeus, bZIPDB, and ORegAnno. The targets of individual transcription factors were counted among the DEGs. To compute the significance of the number of transcription factor target genes, we performed the following procedures: (a) the same number of genes as the number of DEGs was randomly sampled from the whole genome, (b) the target genes of each transcription factor were counted in the randomly sampled genes, (c) this procedure was repeated 100,000 times to generate an empirical null distribution, and (d) the significance of an observed target count in the DEGs was computed using a one‐tailed test with the empirical null distribution. Then, transcription factors whose targets were significantly enriched (*p* < 0.05) by the DEGs were selected as key transcription factors.

### 4.5 Reconstruction of network model

To reconstruct a subnetwork describing interactions between key transcription factors and their targets in IL‐7Rα^low^ EM CD8^+^ T cells, we first selected 98 target genes of the six transcription factors involved in the nine enriched cellular processes from the 774 DEGs. We then built a network model describing the key transcription factor–target interactions and protein–protein interactions among the targets. The transcription factor–target and protein–protein interactions were obtained from six databases including TRED, EEDB, mSigDB, Amadeus, bZIPDB, and OregAnnoand from four databases including HPRD, BioGRID, STRING, and KEGG. The nodes in the network with the same Gene Ontology Biological Processes (GOBPs) and Kyoto Encyclopedia of Genes and Genomes (KEGG) pathway annotations were arranged and grouped into the same network module.

### 4.6 Study cohorts for influenza vaccine responses

We employed three study cohorts to explore the possible relationship of aging signature genes with influenza vaccine responses. The first two cohorts (Yale 1 and Yale 2) were from the study of gene expression microarray analysis of influenza vaccine responses in young and older adults organized by Yale University Health Services during the 2010–2012 seasons (Thakar et al., 2015). The other cohort (Mayo) was from the study of RNA sequencing analysis of PBMCs of older adults (ages 50–74) organized by Mayo Clinic during the 2010–2011 season. The normalized gene expression data and influenza vaccine response information of these cohorts were collected from GEO and the ImmPort website (Ovsyannikova et al., 2016). The data accession IDs of the Yale 1, Yale 2, and Mayo cohorts are GSE59714, GSE95743, and SDY67, respectively. Influenza vaccine responders were defined based on a fourfold or greater increase in the postvaccine hemagglutination inhibition test (HAI) titer to any of three influenza strains in the vaccine (Yale cohorts) or a combination of HAI test results and B‐cell ELISPOT count values at day 28 (Mayo cohort).

### 4.7 qPCR and Western blot analysis

Total RNA was extracted from cells using the RNeasy Plus Midi kit (QIAGEN, Germantown, MD), and cDNA was synthesized. Primer sequences for qPCR are shown in Table S4. Each real‐time PCR was performed on a 10 μl reaction mixture containing cDNA, 2× Brilliant SYBR green master mix (Stratagene), and 3 μM of each primer. The reaction mixture was denatured for 10 min at 94°C and incubated for 40 cycles (denaturing for 15 s at 95°C and annealing and extending for 1 min at 60°C) using the Mx3005P qPCR system (Stratagene). *ACTB* was amplified as an internal control. The relative RNA levels were calculated by the 2^−ΔΔCT^ algorithm.

For Western blot analysis, protein extracts that were separated by SDS–PAGE and transferred onto PVDF membranes were probed with antibodies against MYC (R&D Systems), SATB1 (GeneTex), BATF (Cell Signaling Technology), and beta‐actin (Santa Cruz Biotechnology). The probed membranes were washed and incubated with HRP‐labeled secondary antibodies (Santa Cruz Biotechnology). The bands were then visualized with the Pierce ECL Western blotting substrate (Thermo Scientific).

### 4.8 Statistical analysis

Statistical significance of in vitro and in vivo assays was assessed by the unpaired and paired two‐tailed Student's *t* test or Wilcoxon two‐tailed rank‐sum test as appropriate. Pearson's correlation was used to assess the association between two variables. A permutation test strategy was used to determine the significance of overlapping of the DEGs and age‐associated genes. A total of 100,000 random permuted samples were used to compute the empirical *p* value of the overlapping genes. The logistic regression model was trained by using the gene expression data from the Yale 1 cohort and was validated by using the Yale 2 and Mayo cohorts. Top 10 gene scores were computed using the weighted *z* score method and employed as a predictor variable for influenza vaccine responses in the regression analysis. Area under a receiver operating characteristic curve was used to evaluate the performance of the regression model to predict influenza vaccine responses using the three cohorts. GraphPad Prism (version 7) and MATLAB including the Statistics toolbox (MathWorks) were used for all statistical tests. Cytoscape (v.3.4 http://www.cytoscape.org/) was used to visualize network models.

## CONFLICT OF INTEREST

None declared.

## AUTHOR CONTRIBUTIONS

MS, HJP, MK, SY, and IK designed and performed experiments, analyzed data, and wrote the manuscript. MK and SY designed and performed bioinformatic analysis. JB, SM, ACS, and RRM contributed to experimental design, discussions, and/or provided intellectual input.
