# Supplementary material for: Transcriptomic analysis of human IL‐7 receptor alpha low and high effector memory CD8+ T cells reveals an age‐associated signature linked to influenza vaccine response in older adults
Source: Aging Cell. 2019 May 1;18(4):e12960. doi: 10.1111/acel.12960 (PMC6612637; doi:10.1111/acel.12960)

Supplementary Figure S1

A. Network genes are highly enriched in responder in elderly PBMCs (Yale 1)

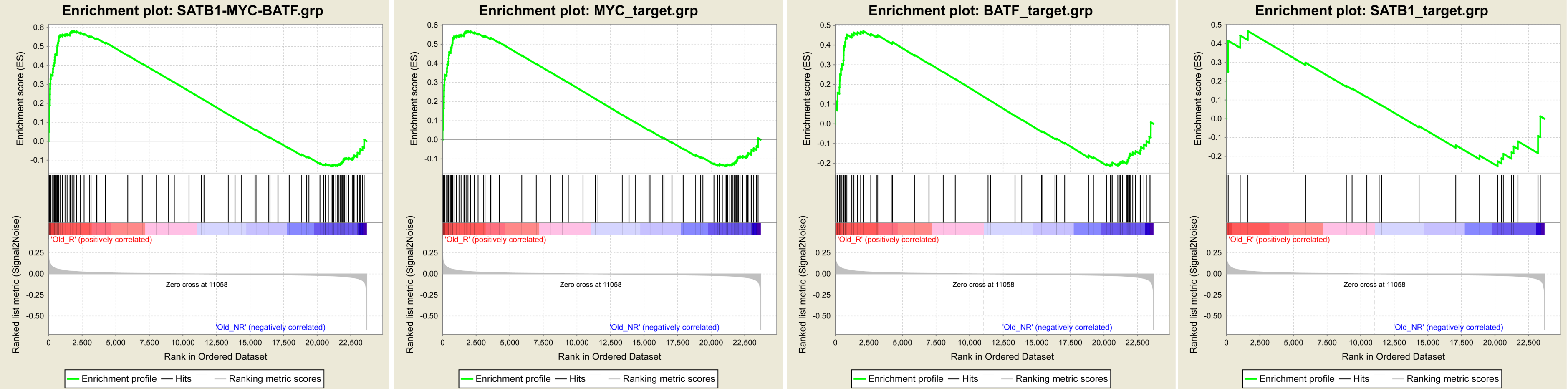

B. Network genes are highly enriched in responder in elderly PBMCs (Yale 2)

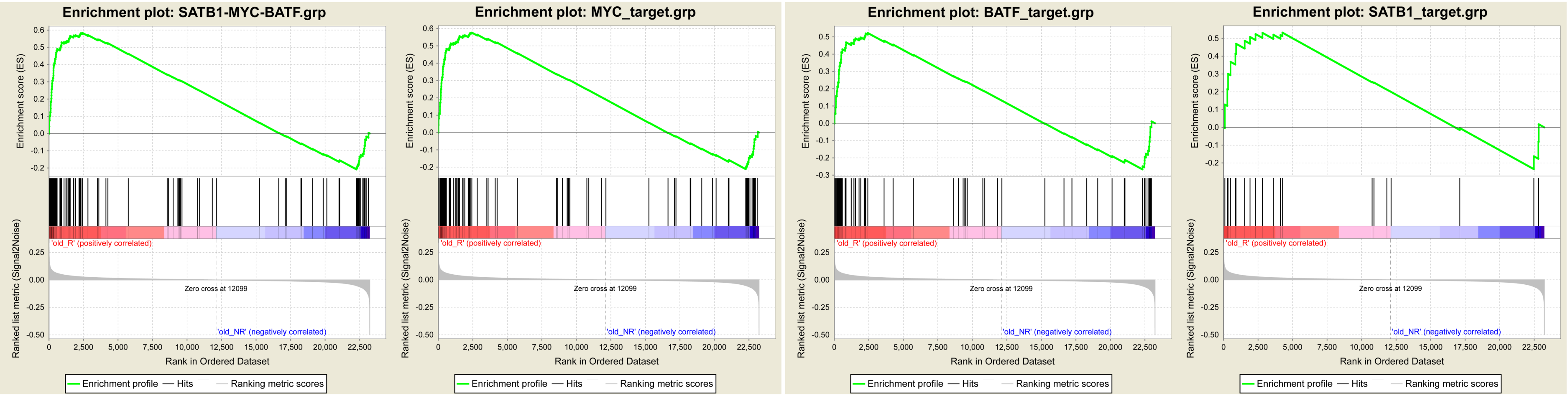

Supplementary Figure S1

C. Network genes are highly enriched in responder in young PBMCs (Yale 1)

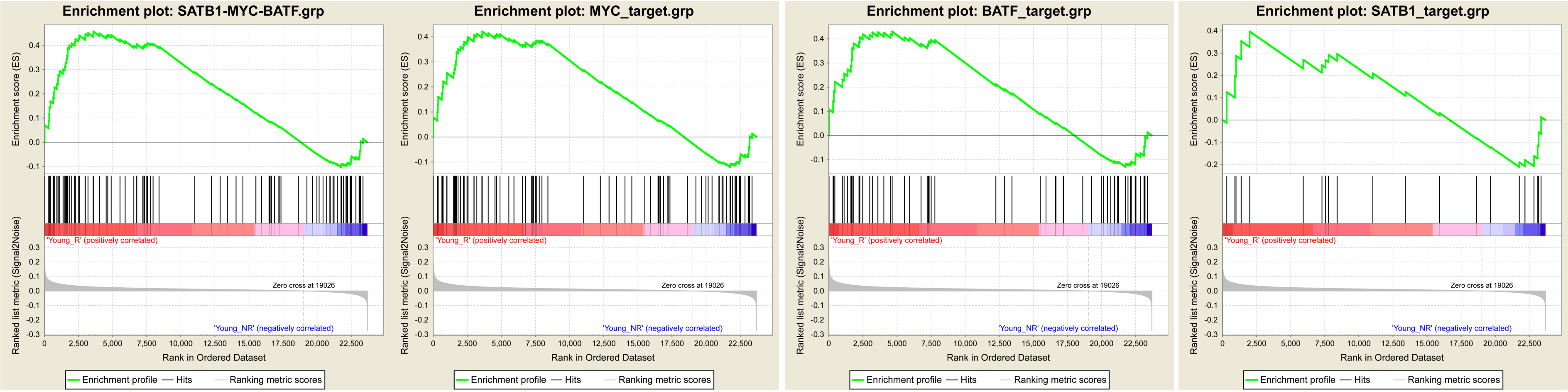

D. Network genes are highly enriched in responder in young PBMCs (Yale 2)

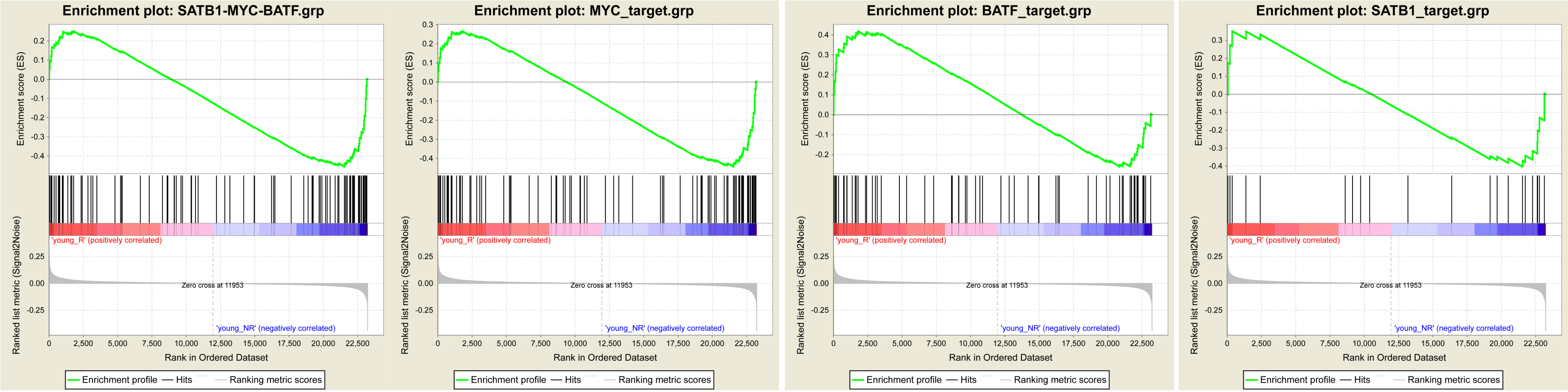

Supplement: Supplementary file 1 [file ACEL-18-e12960-s001.pdf]
