# Supplementary material for: Transcriptomic analysis of human IL‐7 receptor alpha low and high effector memory CD8+ T cells reveals an age‐associated signature linked to influenza vaccine response in older adults
Source: Aging Cell. 2019 May 1;18(4):e12960. doi: 10.1111/acel.12960 (PMC6612637; doi:10.1111/acel.12960)
Supplement: Supplementary file 5 [file ACEL-18-e12960-s005.docx]

**Supplementary Table S4.** Primer sequences for qPCR.

| **Gene** | **Primer sequence** | **Annealing temp (°C)** |
| --- | --- | --- |
| *SATB1* | For: 5'-CAGGAAATGAAGCGTGCTAA-3' | 60°C |
|  | Rev: 5'-GCGTTGCTCTCCTGTTCATA-3' |  |
| *BATF* | For: 5'-TATTGCCGCCCAGAAGAGC-3' | 60°C |
|  | Rev: 5'-GCTTGATCTCCTTGCGTAGAG-3' |  |
| *MYC* | For: 5'-CGACGAGACCTTCATCAAAA-3' | 60°C |
|  | Rev: 5'-TGCTGTCGTTGAGAGGGTAG-3' |  |
| *KLF4* | For: 5'-CGGACATCAACGACGTGAG-3' | 60°C |
|  | Rev: 5'-GACGCCTTCAGCACGAACT-3' |  |
| *NFKB1* | For: 5'-GAAGCACGAATGACAGAGGC-3' | 60°C |
|  | Rev: 5'-GCTTGGCGGATTAGCTCTTTT-3' |  |
| *IRF1* | For: 5'-ATGCCCATCACTCGGATGC-3' | 60°C |
|  | Rev: 5'-CCCTGCTTTGTATCGGCCTG-3' |  |
| *FGFBP2* | For: 5'-TTCCTGCACTATGCGTCCC-3' | 60°C |
|  | Rev: 5'-GGGCTTGATTCCAGTAAGGTTT-3' |  |
| *GZMB* | For: 5'GGGGACCCAGAGATTAAAAA-3' | 60°C |
|  | Rev: 5'-GCTCCAGAGAAGGTGTTTCA-3' |  |
| *GZMH* | For: 5'-ATGAGCACTTTAGCAACCACAC-3' | 60°C |
|  | Rev: 5'-CGGAGTCCCCCTTGAAACC-3' |  |
| *CX3CR1* | For: 5'-TTGCCCTCACCAACAGCAAG-3' | 60°C |
|  | Rev: 5'-AAGGCGGTAGTGAATTTGCAC-3' |  |
| *Actin* | For: 5'-CGTGGACATCCGCAAAGAC-3' | 60°C |
|  | Rev: 5'-TGCATCCTGTCGGCAATG-3' |  |
